# Tibial Tuberosity Avulsion in an Adult Female With Patellar Tendon and Retinacular Repair Enhanced by Platelet-Rich Plasma (PRP) Augmentation: A Case Report

**DOI:** 10.7759/cureus.79953

**Published:** 2025-03-03

**Authors:** Kamil R Jarjess, Saif L Juma, Jamil Haddad, Matthew J Yousif

**Affiliations:** 1 College of Arts and Sciences, Oakland University, Rochester, USA; 2 Medical School, Michigan State University College of Osteopathic Medicine, East Lansing, USA; 3 Department of Orthopedic Surgery, McLaren Macomb Hospital, Mount Clemens, USA; 4 Department of Orthopedic Surgery, Corewell Health William Beaumont University Hospital, Royal Oak, USA

**Keywords:** internal brace construct, patellar tendon rupture, platelet-rich plasma/prp, retinacular repair, tibial tuberosity avulsion

## Abstract

Tibial tuberosity avulsion fractures are rare injuries, most commonly seen in adolescent males during high-energy activities. These fractures are uncommon, specifically in adults, due to the closure of the physis. In particular, skeletally mature female avulsions are rarely reported in the literature. Significant displacement of the tibial tubercle often necessitates surgical management to restore the extensor mechanism and maintain knee stability.

In this case report, we present a 21-year-old female who sustained a tibial tuberosity avulsion fracture with an associated patellar tendon rupture, as well as medial and lateral retinacular injuries following a low-energy fall. Surgical management included a double-row repair technique with SwiveLock anchors (Arthrex, Inc., Naples, FL, USA) and an internal brace construct to reconstruct the extensor mechanism. Simultaneous retinacular repairs were performed, and platelet-rich plasma (PRP) was utilized as an adjunct to enhance soft tissue healing. At six months' follow-up, the patient demonstrated excellent functional recovery, returning to sport-specific activities with no extensor lag, a Lysholm Knee score of 95, and an International Knee Documentation Committee (IKDC) score of 92. In this case, a double-row repair with an internal brace construct was utilized to restore the extensor mechanism, complemented by medial and lateral retinacular repair to enhance knee stability. Additionally, PRP was incorporated as an adjunct to support soft tissue healing.

## Introduction

Tibial tuberosity avulsion fractures are rare, accounting for less than 3% of epiphyseal injuries in children and even fewer cases in adults [1,2]. These fractures primarily affect athletic males, approaching skeletal maturity, a period when the tubercle apophysis is especially prone to avulsion due to its mechanical vulnerability [3,4].

The tibial tubercle, located about 3 cm below the tibial plateau, aligns with the medial patella when the knee is flexed and the lateral patella when extended [5]. It serves as the attachment site for the patellar tendon, which is the terminal extension of the quadriceps muscle. This injury can occur through two mechanisms: a forceful quadriceps contraction during knee extension, as in jumping, or eccentric knee flexion against a strongly contracted quadriceps, such as landing from a jump [6]. Associated injuries may involve patellar and quadriceps tendon avulsions, damage to the medial and lateral retinacular structures, tears of the collateral and cruciate ligaments, and meniscal injuries [7]. Initial assessment of tibial tubercle fractures commonly involves radiographs of the knee, including anterior-posterior (AP) and lateral views, with the lateral view providing the clearest depiction of the injury [3]. Tibial tubercle avulsion fractures are categorized based on the Ogden Classification [8]. Nonoperative treatment with a long leg cast in extension for six weeks is appropriate for Type I tibial tubercle avulsions or minimally displaced fractures (<2 mm) [4]. Operative management with open reduction internal fixation (ORIF) is necessary for Type II-IV fractures to achieve optimal joint surface reduction and evaluate intra-articular pathology. Type V (periosteal sleeve) fractures require surgical repair with soft tissue reconstruction to restore the extensor mechanism and reduce the risk of refracture [9].

A tibial tubercle fracture requiring simultaneous medial and lateral retinacular repair is exceedingly rare in adult females, with only limited reports in the literature. Here, we present the case of a 21-year-old female who sustained a tibial tuberosity avulsion fracture after a low-energy fall, landing with her knee in a flexed position while attempting to stabilize herself. Surgical intervention involved fixation, utilizing a double-row repair technique with an internal brace construct. Platelet-rich plasma (PRP) was used as an adjunct to promote soft tissue healing. Notably, no risk factors were identified in the patient that would predispose her to the occurrence of the fracture.

## Case presentation

A 21-year-old female presented with acute right knee pain and swelling following a non-contact injury. She described a sudden onset of symptoms while ambulating, without any preceding trauma, instability, or systemic complaints. Her medical history was unremarkable, with no prior musculoskeletal injuries, chronic illnesses, or family history.

On examination, there was significant knee swelling with localized tenderness over the tibial tubercle. A palpable defect was noted at the inferior pole of the patella, raising suspicion for extensor mechanism disruption. Passive knee flexion was preserved, but the extensor mechanism was clearly disrupted, with a lack of active knee extension. Neurovascular examination was intact, with normal capillary refill, dorsalis pedis and posterior tibial pulses, and symmetric sensory function.

**Figure 1:**
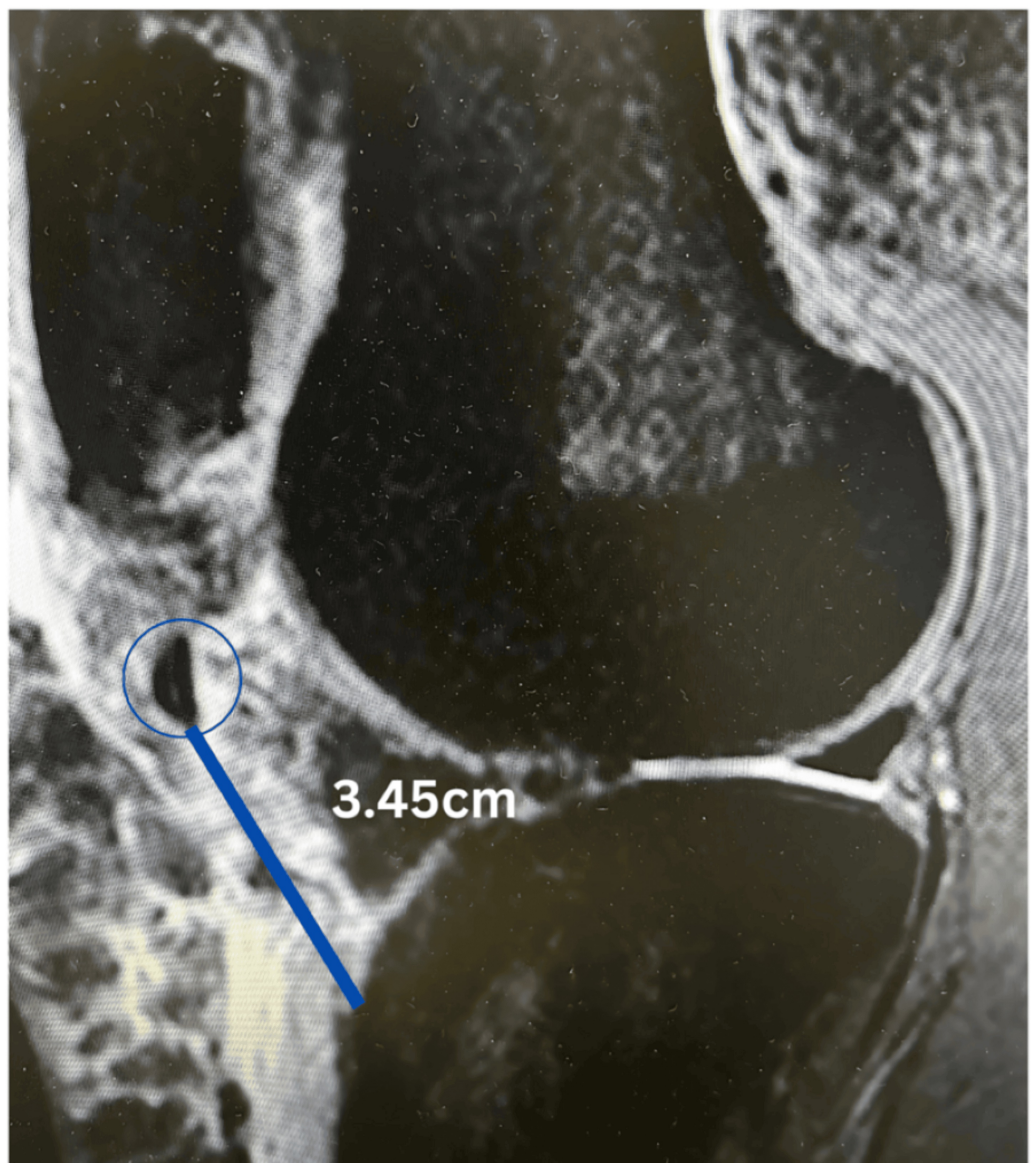
Initial MRI of the right knee MRI demonstrating a tibial tuberosity avulsion fracture (circled in blue) with retraction measured at approximately 3.45 cm.

Plain radiographs revealed a small avulsion of the tibial tuberosity that was retracted to the level of the distal femur. An MRI confirmed a complete patellar tendon rupture at its tibial insertion, with associated avulsion of the tibial tubercle measuring approximately 5 mm in height, 2 mm in depth, and 3 mm in width (Figure 1). The extensor mechanism was disrupted, necessitating surgical intervention for optimal, speedy functional recovery.

A midline longitudinal incision was made over the anterior knee, extending from the distal patella to the proximal tibia. Dissection was carried through the subcutaneous tissue, revealing hemorrhagic paratenon, which was carefully split to expose the ruptured patellar tendon. The avulsed tibial tubercle fragment was identified.

No screws or metal hardware was used in the fixation, as the bony fragment was too small to allow for drilling. The tendon and osseous fragment were reduced to their native footprint on the tibial tubercle. The medial row fixation was achieved by securing the Krackow suture limbs into two 4.75 mm SwiveLock anchors (Arthrex, Inc., Naples, FL, USA) placed proximally on the tibial plateau. The sutures were crisscrossed to provide compression and stabilization over the avulsion site, ensuring anatomic restoration of the extensor mechanism.

**Figure 2:**
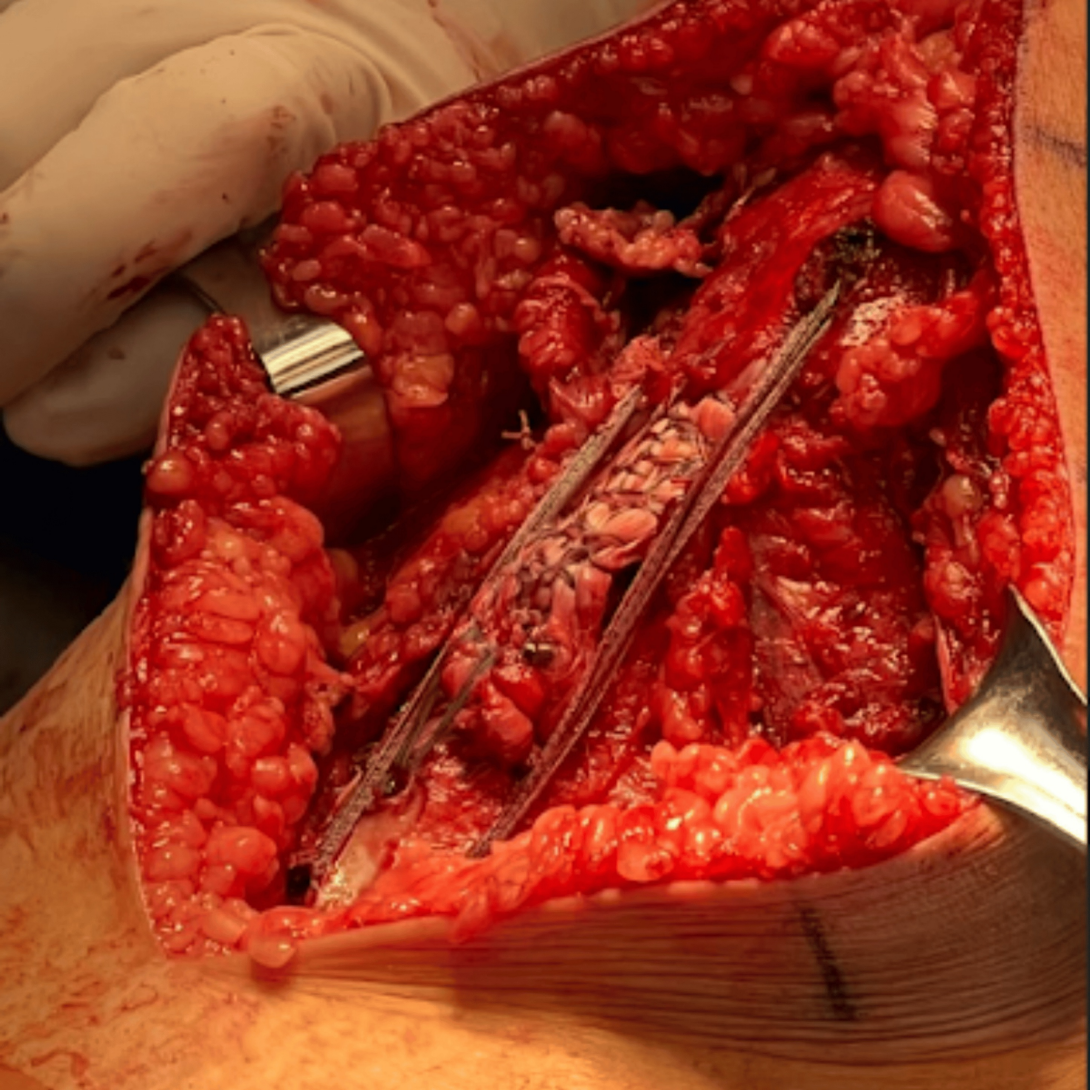
Intraoperative view of double-row fixation for patellar tendon repair Double-row fixation construct with Krackow suture limbs securing the patellar tendon repair.

To reinforce the repair, two additional 4.75 mm SwiveLock anchors were placed distal to the avulsion site in the prepared tibial footprint. The suture tapes from the medial row were then tensioned and shuttled through these distal anchors, creating a double-row fixation construct (Figure 2).

This method provided enhanced compression across the tibial tubercle fracture and improved tendon apposition. An internal brace augmentation was performed for secondary stabilization. With the knee positioned at 30° of flexion to prevent over-tensioning, a 4.75 mm SwiveLock anchor was placed in the patella. A suture tape was routed along the lateral edge of the patellar tendon and secured into a second SwiveLock anchor, lateral to the tibial tubercle. Similarly, a medial internal brace was established with a SwiveLock anchor placed at the medial patellar tendon edge, securing into another SwiveLock just medial to the tibial tubercle.

The knee was taken through a range of motion from 0° to 90°, demonstrating a robust patellar tendon repair and a properly tensioned internal brace that augmented the repair. The internal brace was applied using the Arthrex standard technique at 30° of flexion, ensuring the patellar tendon was not over-tensioned. Four SwiveLock anchors were used, two in the patella and two positioned medially and laterally to the previously placed SwiveLock anchors in the tibial tubercle.

**Figure 3:**
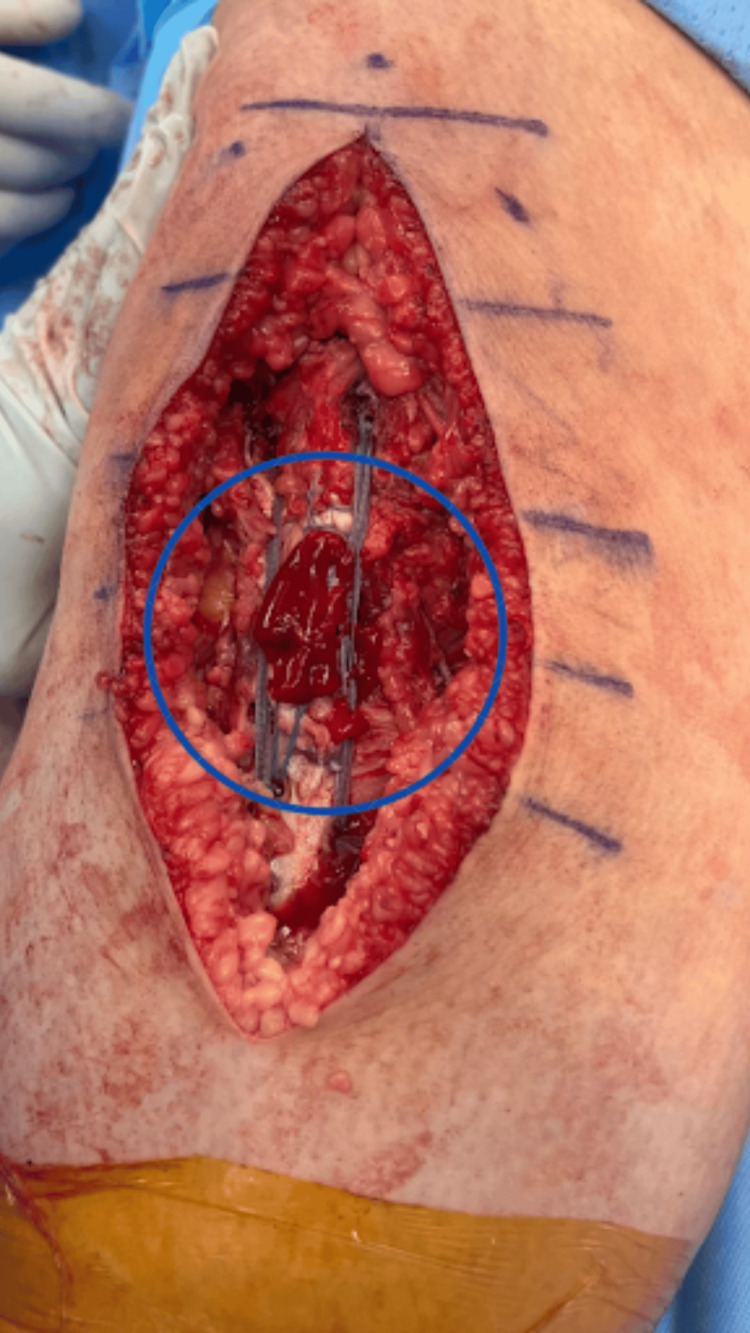
Platelet-rich plasma (PRP) application over patellar tendon repair Intraoperative application of platelet-rich plasma (PRP), circled in blue, superficial to the patellar tendon repair site to enhance soft tissue healing.

PRP, harvested at the beginning of the case and mixed with 10 mL of sterile saline and 10,000 units of calcium chloride, was applied to the surgical site for activation of platelets and the polymerization of fibrin. Leukocyte-rich PRP was placed over the patellar tendon following thorough irrigation (Figure 3).

**Figure 4:**
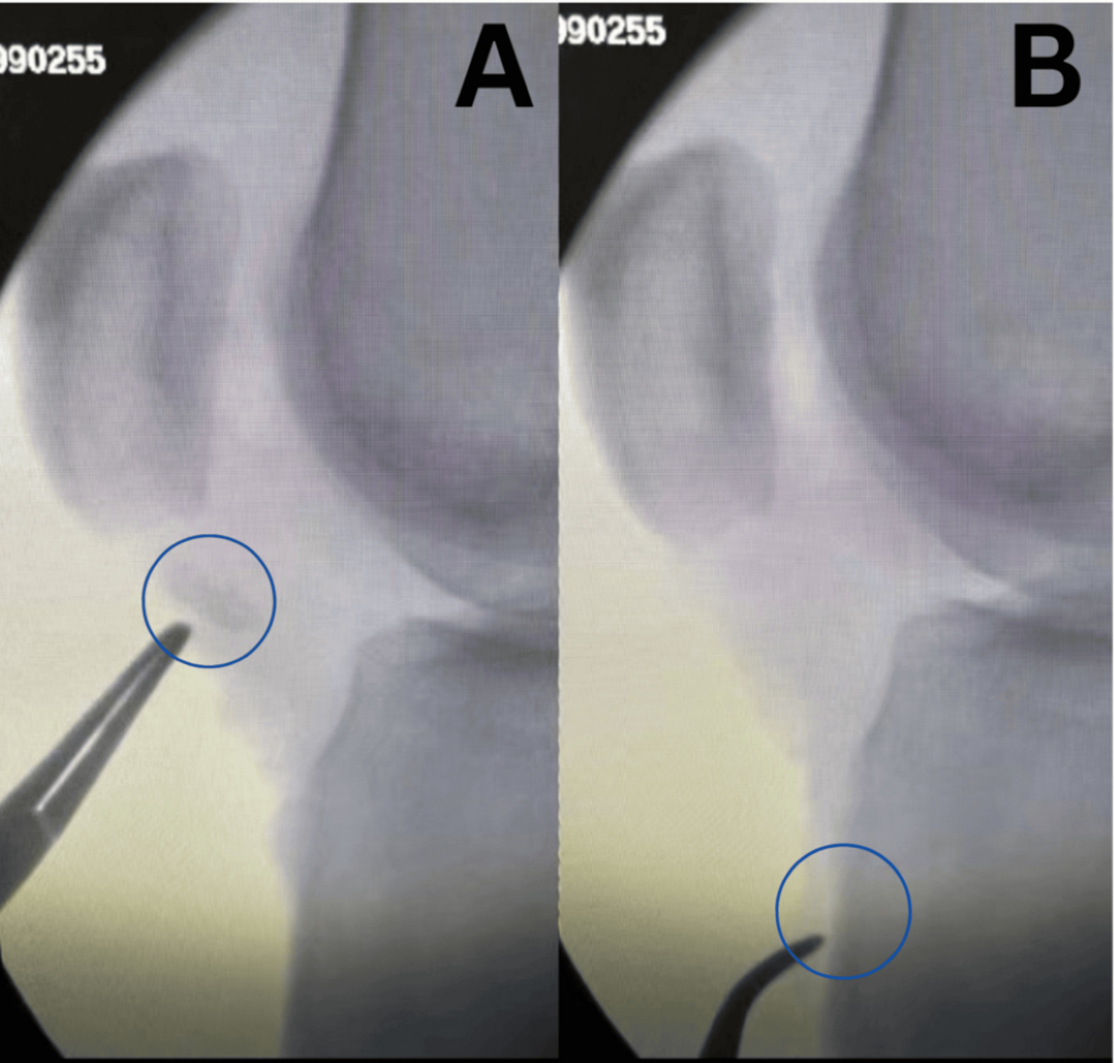
Intraoperative radiographs of tibial tubercle avulsion reduction and fixation Intraoperative radiographs demonstrating intraoperative reduction of the osseous fragment. Image A depicts the condition before fixation of the displaced tibial tubercle avulsion, while Image B illustrates the restored alignment following fixation.

Final intraoperative X-rays demonstrated an anatomic reduction of the patellar height, consistent with a robust patellar tendon repair. The tourniquet time was approximately 120 minutes. The patient was safely extubated and transferred to recovery (Figure 4).

Postoperatively, the patient followed a comprehensive rehabilitation protocol divided into four progressive phases: immobilization (0-2 weeks), range of motion (3-6 weeks), strengthening (7-12 weeks), and sport-specific functional rehabilitation (4-6 months). By 12 weeks, she achieved full weight-bearing without a brace, regained full knee flexion, and demonstrated a symmetric gait pattern. At six months, the patient returned to sport-specific activities with no extensor lag and excellent functional outcomes, evidenced by a Lysholm Knee score of 95 (excellent category) and an International Knee Documentation Committee (IKDC) score of 92, indicating near-complete recovery of knee function and stability.

## Discussion

Tibial tuberosity avulsion fractures are predominantly observed in adolescent males, with a reported mean age of injury of 13 years and 8 months [10]. However, this injury is exceptionally rare in adults, particularly in women, making the presentation of this case particularly unique. While tibial tuberosity avulsion fractures are typically seen in the context of high-energy trauma, such as significant sports injuries, our patient sustained the fracture following a low-energy fall [11]. This highlights an atypical mechanism of injury for this fracture type, especially in an already uncommon demographic. Such fractures usually result from a sudden contraction of the quadriceps during knee extension, or passive knee flexion against rapid quadriceps contraction, often observed during activities like jumping or stabilizing the knee in flexion [12].

In their systematic review, Pretell-Mazzini et al. found that tibial tuberosity avulsion fractures are significantly more common in the left knee (59%) and predominantly affect males (97%). This male predominance is attributed to several factors, including greater participation in high-impact sports, higher quadriceps muscle strength in men, and hormonal factors, with later physeal closure in males [13]. These factors collectively increase the risk of extensor mechanism failure, leading to tibial tuberosity fractures [14]. While these injuries are most often associated with high-impact activities, cases have also been reported in low-impact activities, such as running, suggesting that additional factors may predispose individuals to physeal line weakness [15].

Management of this injury required careful consideration of both the fracture and the associated soft tissue damage. Surgical intervention was necessary due to the displacement of the fracture and the need to restore the extensor mechanism. This case represents an Ogden Type IIIb tibial tubercle avulsion fracture, characterized by intra-articular extension with significant displacement and associated patellar tendon rupture. The radiographic findings of a small avulsed tibial tubercle fragment, retracted proximally to the distal femur, along with MRI confirmation of complete patellar tendon rupture at its tibial insertion, align with this classification. Type IIIb fractures necessitate surgical intervention due to extensor mechanism disruption, requiring both fracture fixation and patellar tendon repair. The fractured tibial tubercle component was identified, which had been avulsed due to the significant force applied through the patellar tendon and quadriceps mechanism. A Krackow stitch was used on the patellar tendon, providing strong tendon-to-tendon fixation. This suture technique creates multiple suture limbs that are passed through the tendon, allowing for better load distribution across the repair site [16]. Two SwiveLock anchors were then utilized to secure the patellar tendon near its insertion on the tibial tubercle. The free limbs of the sutures were passed back through the tendon using a free needle, creating a medial row suture construct to further secure the tendon. In addition to the tendon repair, a double-row repair technique with an internal brace construct was employed, ensuring robust fixation and stability, as demonstrated in the literature. Simultaneous medial and lateral retinacular repair was performed to address associated soft tissue injuries and enhance knee stability.

In addition to the primary repair techniques, PRP was utilized to enhance healing, particularly considering the complex nature of the injury. While PRP’s full potential in orthopedic surgery is still under investigation, studies suggest it may facilitate cellular regeneration and modulate inflammation, potentially improving outcomes in soft tissue injuries [17,18]. Theoretically, leukocytes within PRP have been proposed to possibly inhibit bacterial growth, reducing the risk of postoperative infection and promoting healing by enhancing soft tissue regeneration [19]. Given the significant soft tissue involvement, particularly with the retinacular repair, PRP was used as adjunct therapy to optimize the healing environment. The regenerative properties of PRP may have contributed to both tendon and retinacular repair, facilitating recovery in this rare and complicated case.

This case highlights the importance of considering alternative treatment options, such as the use of PRP, in the management of rare orthopedic injuries. Future research may provide further insight into optimizing surgical techniques and adjunct therapies in similar complex cases. The combination of surgical repair techniques and regenerative medicine could become a valuable approach in managing tibial tuberosity avulsion fractures, particularly in adults and females, where such injuries are infrequent.

## Conclusions

We present a rare case of a 21-year-old skeletally mature female with a tibial tubercle avulsion fracture from a low-energy fall, caused by eccentric quadriceps contraction and excessive patellar tendon tension. Surgical management involved a double-row repair with an internal brace construct to restore the extensor mechanism, along with medial and lateral retinaculum repair for enhanced knee stability. PRP was used as an adjunct to promote soft tissue healing. At six months, the patient demonstrated excellent functional recovery, returning to sport-specific activities with no extensor lag, a Lysholm Knee score of 95, and an IKDC score of 92.
